# Synthesis, Ligational and Biological Properties of Cobalt(II), Copper(II), Nickel(II) and Zinc(II) Complexes With Pyrazinedicarboxaimide Derived Furanyl, Thienyl and Pyrrolyl Compounds

**DOI:** 10.1155/MBD.1998.347

**Published:** 1998

**Authors:** Zahid H. Chohan, S. K. A. Sherazi, M. Praveen, M. S. Iqbal

**Affiliations:** 1 Department of Chemistry Islamia University Bahavalpur Pakistan; 2 Department of Chemistry Washington University St. Louis 63130 USA; 3 Himont Pharmaceutical and Chemical Laboratories Lahore Pakistan

## Abstract

Preparation, ligational and biological properties of some pyrazinedicarboxaimide derived furanyl, thienyl and pyrrolyl compounds with Co(ll), Cu(ll), Ni(ll) and Zn(ll) metals are described. Magnetic moments, electronic, infrared, nuclear magnetic resonance spectra and elemental analysis data indicate that co-ordination of the ligands with the metal ions take place through the pyrazine ring nitrogen, azomethine nitrogen and heteroatom of heterocyclic ring system. The compounds are all novel and are proposed to possess an octahedral geometry for Co(ll) and Ni(ll), and a distorted octahedral geometry for Cu(ll) and Zn(ll) complexes. The comparative biological properties of the title ligands and their metal chelates against different bacterial species are also described.


### SYNTHESIS, LIGATIONAL AND BIOLOGICAL PROPERTIES OF COBALT(II), COPPER(II), NICKEL(II) AND ZINC(II) COMPLEXES WITH PYRAZINEDICARBOXAIMIDE DERIVED FURANYL, THIENYL AND PYRROLYL COMPOUNDS

Zahid H. Chohan*[1], S. K. A. Sherazi[1], M. Praveen[2] and M. S. Iqbal[3]

[1] Department of Chemistry, Islamia University, Bahawalpur, Pakistan
[2] Department ofChemistry, Washington University, St. Louis 63130, USA
[3] Himont Pharmaceutical and Chemical Laboratories, Lahore, Pakistan

### ABSTRACT

Preparation, ligational and biological properties of some pyrazinedicarboxaimide derived furanyl, thienyl and pyrrolyl compounds with Co(II), Cu(II), Ni(II) and Zn(II) metals are described. Magnetic moments, electronic, infrared, nuclear magnetic resonance spectra and elemental analysis data indicate that co-ordination of the ligands with the metal ions take place through the pyrazine ring nitrogen, azomethine nitrogen and heteroatom of heterocyclic ring system. The compounds are all novel and are proposed to possess an octahedral geometry for Co(II) and Ni(II), and a distorted octahedral geometry for Cu(II) and Zn(II) complexes. The comparative biological properties of the title ligands and their metal chelates against different bacterial species are also described.

### INTRODUCTION

Several studies have been reported[1-5] on the complexes of metals with pyridine and pyrazine derivatives having active donor substituents e.g., pyridine-2-carboxamide[1], aminopyrazine[6,7], pyrazinyl ketone[8] and pyrazinecarboxylic acid[9]. In spite of their interesting co-ordination chemistry, their pronounced biologically active nature, which came into light also, attracted us[10-13] to synthesize various pyridinoyl- and pyrazinoyl hydrazine derived compounds and to investigate their co-ordination chemistry and biological properties. In continuation to the same, we now wish to report a novel class of pyrazinedicarboxaimide derived compounds (Figure 1) and study their ligational and biological behavior towards cobalt(II), copper(II), nickel(II) and zinc(II) metals.

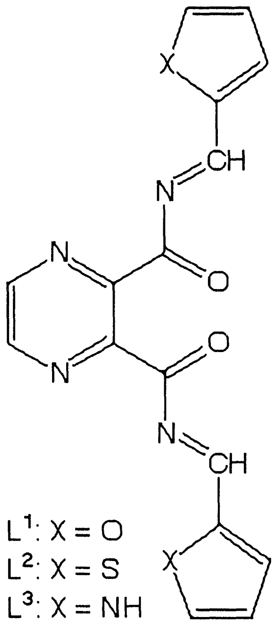

Figure 1 Structure of the Ligand

All the prepared ligands and their complexes have been characterized by their conductance and magnetic measurements, infrared, electronic and NMR spectral studies and elemental analysis data. On the basis of these investigations, ultimate geometry of these complexes have been proposed to be octahedral for Co(II) and Ni(II), and distorted octahedral for Cu(II) and Zn(II) complexes.

**Table 1 Physical, Spectral and Analytical Data of the Ligands**

| Schiff base/ Mol. Form. | M.P. (°C) | IR ($cm^{-1}$) | Calc (Found %) C | H | N |
|---|---|---|---|---|---|
| **L1** $C_{16}H_{10}N_4O_4$ [322.16] | 173 | 2035, 1945, 1670, 1625, 1575, 1515, 1350, 1060, 875, | 59.6 (59.8) | 3.1 (2.9) | 17.4 (16.9) |
| **L2** $C_{16}H_{10}N_4O_2S_2$ [354.28] | 187 | 2035, 1945, 1670, 1625, 1575, 1515, 1350, 1063, 875 | 54.2 (54.6) | 2.8 (3.9) | 15.8 (15.6) |
| **L3** $C_{16}H_{12}N_6O_2$ [428.52] | 192 | 3045, 2818, 2035, 1945, 1670, 1625, 1575, 1515, 1355, 1062, 345 | 44.8 (45.0) | 2.8 (2.7) | 44.9 (44.5) |

**Table 2 $^1$H NMR and $^{13}$C NMR Data of the Ligands**

| Ligand | $^1$H NMR (DMSO-$d_6$) (ppm) | $^{13}$C NMR (DMSO-$d_6$) (ppm) |
|---|---|---|
| L1 | 4.54-4.56 (m, 2H, furanyl), 4.68 (dd, 2H, furanyl), 5.88 (s, 2H, furanyl), 6.13 (s, 2H, azomethine), 7.91-7.94(m, 1H, pyrazine), 8.14-8.18 (m, 1H,pyrazine) | 104.71 ($C_3$), 112.24 ($C_7$), 121.36 ($C_5$), 123.22 ($C_4$), 124.1 ($C_6$), 126.2 ($C_2$), 152.31 (C=N), 187.38 (C=O). |
| L2 | 4.55-4.57 (m, 2H, thienyl), 4.71 (dd, 2H, thienyl), 5.86(s,2H, thienyl), 6.15 (s, 2H, azomethine), 7.94 - 7.96 (m, 1H, pyrazine), 8.14-8.16 (m, 1H, pyrazine) | 104.6 ($C_3$), 112.21 ($C_7$), 121.18 ($C_5$), 123.21 ($C_4$), 124.53 ($C_6$), 126.08 ($C_2$), 153.13 (C=N), 186.92 (C=O). |
| L3 | 4.53-4.55 (m, 2H, pyrrol), 4.66 (dd, 2H, pyrrol), 5.85 (s, 2H, pyrrol), 6.15 (s, 2H, azomethine), 7.93-7.95 (m, 1H, pyrazine), 8.13-8.16 (m, 1H, pyrazine), 8.48 (s, 1H, NH,), | 104.11 ($C_3$), 112.56 ($C_7$), 121.3 ($C_5$), 123.46 ($C_4$), 124.41 ($C_6$), 126.82 ($C_2$), 153.0 (C=N), 188.27 (C=O). |

### EXPERIMENTAL

### Material and Methods

$^1$H, and $^{13}$C solution nmr spectra were recorded on a Brucker 250 MHz instrument, at the Department of Chemistry, University of Aberdeen (U.K). $^{13}$C-NMR spectra were recorded with H spin decoupling. The IR spectra were obtained using an ATI Matheson ICON, FTIR spectrophotometer. Melting points were recorded on a Kofler Hostage and are uncorrected. Butterworth Laboratories Ltd, Middlesex, (U.K), carried out analyses of C, H and N. Electronic spectra were recorded on Hitachi double-beam U-2000 spectrophotometer using glass cells of 1 cm thickness. Conductance was measured on a conductance meter YSI model-32 and magnetic susceptibility on a Gouys balance. All chemicals used were Analar grade. Metals were used as their chlorides. Pyrazine-2,3-dicarboxamide was obtained from Aldrich Chemical Company.

### Preparation of the Ligands

**N, N(-Bis(2-furanylmethylene) pyrazine-2,3-dicarboxaimide (L1)**

Furan-2-aldehyde (1.6 mL, 1.92 g, 0.02 mol) in absolute ethanol (35 mL) was added to a magnetically stirred n-butanol solution (25 mL) of pyrazine-2,3-dicarboxamide (0.01 mol). Then 5-10 drops of concentrated sulfuric acid were added and mixture refluxed for 4 h. The mixture on cooling immediately gave a solid product, which was filtered, washed with n-butanol (2x10 mL) and dried. It was then crystallized in hot aqueous n-butanol (50 %) to yield **L1** (1.65 g) (67 %)

**N, N-[Bis(2-thienylmethylene) pyrazine-2,3-dicarboxaimide] ($L^2$)**
Thiophene-2-aldehyde (1.84 mL, 2.24 g, 0.02 mol) in absolute ethanol (30 mL) was added to a stirred n-butanol solution (25 mL) of pyrazine-2,3-dicarboxamide (0.01 mol). Then 5-10 drops of concentrated sulfuric acid were added and mixture refluxed for 4 h. It was then cooled and solid product thus obtained was filtered, washed with n-butanol (2x15 mL) and dried. On crystallization in hot aqueous n-butanol (50 %) gave 1.57g (62 %).
**N, N-[Bis(2-pyrrolmethylene) pyrazine-2,3-dicarboxaimide] ($L^3$)**
Pyrrol-2-aldehyde (1.9 g, 0.02 mol) in absolute ethanol (35 mL) was added to a solution of pyrazine-2,3-dicarboxamide (0.01 mol) in n-butanol (30 mL). Then 5-10 drops of concentrated sulfuric acid were added in it and mixture refluxed for 4 h. This reaction mixture, on cooling gave a solid product which was filtered, washed with n-butanol (2x15 mL) and dried. The solid thus obtained was crystallized in hot aqueous n-butanol (50 %) to give **$L^3$** (1.58 g) (59 %).

**Table 3 Physical Data of the Metal Chelates**

| No | Metal chelate/ Mol. Formula | M.P. (°C) (decomp) | B.M ($\mu_{eff}$) | Calc (Found)% C H N |
|---|---|---|---|---|
| 1 | $[Co(L^1)_2(Cl)_2]$ $C_{32}H_{20}CoCl_2N_8O_8$ [774.15] | 216-218 | 4.68 | 49.6 2.6 14.5 (49.5) (2.5) (14.6) |
| 2 | $[Co(L^2)_2(Cl)_2]$ $C_{32}H_{20}CoCl_2N_8O_4S_4$ [838.39] | 221-222 | 4.45 | 45.8 2.4 13.4 (45.8) (2.6) (13.4) |
| 3 | $[Co(L^3)_2(Cl)_2]$ $C_{32}H_{24}CoCl_2N_{12}O_4$ [770.15] | 209-211 | 4.56 | 49.9 3.1 21.8 (50.1) (2.9) (21.8) |
| 4 | $[Ni(L^1)_2(Cl)_2]$ $C_{32}H_{20}NiCl_2N_8O_8$ [774.15] | 230-231 | 2.88 | 49.6 2.6 14.5 (49.7) (2.8) (14.6) |
| 5 | $[Ni(L^2)_2(Cl)_2]$ $C_{32}H_{20}NiCl_2N_8O_4S_4$ [838.15] | 233-235 | 3.14 | 45.8 2.4 13.4 (45.9) (2.2) (13.2) |
| 6 | $[Ni(L^3)_2(Cl)_2]$ $C_{32}H_{24}NiCl_2N_{12}O_4$ [769.91] | 212-214 | 2.92 | 49.9 3.1 21.8 (50.2) (2.3) (21.9) |
| 7 | $[Cu(L^1)_2(Cl)_2]$ $C_{32}H_{20}CuCl_2N_8O_8$ [778.76] | 238-241 | 1.55 | 49.4 2.6 14.4 (49.1) (2.4) (14.1) |
| 8 | $[Cu(L^2)_2(Cl)_2]$ $C_{32}H_{20}CuCl_2N_8O_4S_4$ [843.0] | 231-233 | 1.82 | 45.6 2.4 13.3 (45.7) (2.5) (13.2) |
| 9 | $[Cu(L^3)_2(Cl)_2]$ $C_{32}H_{24}CuCl_2N_{12}O_4$ [774.76] | 228-230 | 1.76 | 49.6 3.1 21.7 (49.7) (2.2) (21.9) |
| 10 | $[Zn(L^1)_2(Cl)_2]$ $C_{32}H_{20}ZnCl_2N_8O_8$ [780.60] | 222-224 | dia | 49.2 2.6 14.3 (48.9) (2.8) (14.4) |
| 11 | $[Zn(L^2)_2(Cl)_2]$ $C_{32}H_{20}ZnCl_2N_8O_4S_4$ [844.84] | 211-214 | dia | 45.5 2.4 13.3 (45.4) (2.5) (13.1) |
| 12 | $[Zn(L^3)_2(Cl)_2]$ $C_{32}H_{24}ZnCl_2N_{12}O_4$ [564.49] | 202-204 | dia | 49.5 3.1 21.6 (49.5) (3.1) (21.6) |

**Preparation of the Metal Complexes**

To a hot n-butanol solution (30 mL) of the ligand (0.01 mol) was added an ethanolic solution (25 mL) of the respective metal(II) chloride salt (0.01 mol). The mixture was refluxed for 2 h. The resulting mixture was cooled, filtered and reduced to half of its volume (20 mL). The concentrated solution so obtained was left overnight at room temperature which resulted in the formation of a solid product. The product thus formed was filtered, washed with n-butanol (2x10 mL), then with ethanol (2x10 mL) followed by ether (2x20 mL) and dried. Crystallization in hot aqueous n-butanol (50 %) gave **1** (0.67 %), **2** (65 %), **3** (58 %), **4** (67 %), **5** (65 %), **6** (69 %), **7** (55 %), **8** (60 %), **9** (65 %), **10** (58 %), **11** (68 %) and **12** (65 %).

**Table 4 Spectral and Analytical Data of the Metal Chelates**

| No | IR ($cm^{-1}$) | $\lambda_{max}$ ($cm^{-1}$) |
|---|---|---|
| 1 | 1670 (C=O), 1630 (C=N), 1525, 620 (pyrazine ring), 445 (M-O) | 29205, 18352, 8165 |
| 2 | 1670 (C=O), 1632 (C=N), 1530, 625 (pyrazine ring), 375 (M-S) | 30265, 19225, 9740 |
| 3 | 1670 (C=O), 1635 (C=N), 1533, 625 (pyrazine ring), 510 (M-N) | 31260, 18550, 8595 |
| 4 | 1670 (C=O), 1635 (C=N), 1525, 625 (pyrazine ring), 445 (M-O) | 26375, 19185, 9690 |
| 5 | 1670 (C=O), 1630 (C=N), 1530, 620 (pyrazine ring), 375 (M-S) | 27650, 16220, 9455 |
| 6 | 1670 (C=O), 1635 (C=N), 1532, 625 (pyrazine ring), 515 (M-N) | 26873, 17523, 10112 |
| 7 | 1670 (C=O), 1630 (C=N), 1537, 630 (pyrazine ring) 455 (M-O) | 23418, 29450 |
| 8 | 1670 (C=O), 1635 (C=N), 1535, 625 (pyrazine ring), 382 (M-S) | 23155, 30675 |
| 9 | 1670 (C=O), 1635 (C=N), 1537, 625 (pyrazine ring) 525 (M-N) | 23270, 29888 |
| 10 | 1670 (C=O), 1635 (C=N), 1535, 633 (pyrazine ring), 445 (M-O) | 13220 |
| 11 | 1670 (C=O), 1630 (C=N), 1535, 630 (pyrazine ring), 380 (M-S), 365 (N-N) | 13125 |
| 12 | 1670 (C=O), 1635 (C=N), 1538, 635 (pyrazine ring), 525 (M-N) | 13514 |

**Antibacterial Studies**

**Preparation of Discs.**

The ligand/complex (30 (g) in DMF (0.01mL) was applied on a paper disc, [prepared from blotting paper (3 mm diameter)] with the help of a micropipette. The discs were left in an incubator for 48 h at 37° C and then applied on the bacteria grown agar plates.

**Preparation of Agar Plates.**

Minimal agar was used for the growth of specific bacterial species. For the preparation of agar plates for *Escherichia coli*, MacConkey agar (50 g), obtained from Merck Chemical Company, was suspended in freshly distilled water (1 L). It was allowed to soak for 15 minutes and then boiled on a water bath until the agar was completely dissolved. The mixture was autoclaved for 15 minutes at 120° C and then poured into previously washed and sterilized Petri dishes and stored at 40° C for inoculation.

**Procedure of Inoculation.**

Inoculation was done with the help of a platinum wire loop which was made red hot in a flame, cooled and then use for the application of bacterial strains.

**Application of Discs.**

A sterilized forceps was used for the application of paper disc on the already inoculated agar plates. When the discs were applied, they were incubated at 37° C for 24 h. The zone of inhibition was then measured (in diameter) around the disc.

### RESULTS AND DISCUSSION

The ligands were prepared by adopting the same procedure as reported earlier by us[14,15]. The structural determination of these ligands was done with the help of their IR, $^1$H-NMR, $^{13}$C-NMR and microanalytical data (Table 1 & 2). The tentative assignment of some of the important IR bands of ligand is recorded in Table 1. The IR spectra of the free ligands show characteristic absorption bands at 1670, 1625, 1575 and 1515 $cm^{-1}$. These bands are assigned to ν(C=O), ν(C=N) and ν(C=C) pyrazine ring stretches, respectively. The disappearance of the band at 3260 $cm^{-1}$ due to ν(NH) and appearance of a new band at 1625 $cm^{-1}$ due to azomethine linkage confirmed the formation of ligands $L^1$, $L^2$ and $L^3$. The $^1$H-NMR and $^{13}$C-NMR spectra (Table 2) also display signals assignable to the azomethine and other expected ring protons and carbons assigned for the proposed structures of ligands. Carbon-13 was assigned by mainly comparing the values with the reported[16] values. Also, the microanalytical data (Table 1) was found to be in agreement with the molecular structure of the title ligands. Metal complexes of these ligands were prepared by stoichiometric reaction of the respective ligands in the molar ratio M:L = 1:2 (Equation 1).

$$2\,L + MCl_2 \rightleftharpoons [M(L)_2]Cl_2 \qquad \text{(Equation 1)}$$

$L=L^1$, $L^2$ and $L^3$

M=Co(II), Cu(II), Ni(II) and Zn(II)

All the complexes **(1-12)** are air and moisture stable solids. They are soluble in DMF, DMSO and water and insoluble in other solvents. The conductivity measurement of these complexes (22-28 $ohm^{-1}$. $cm^2$. $mol^{-1}$) in DMF shows that they are all non-electrolyte[17,18].

**Table 5 Antibacterial Activity Data**

| Ligand/Chelate | Microbial Species a | b | c | d |
|---|---|---|---|---|
| $L^1$ | ++ | + | + | ++ |
| $L^2$ | + | ++ | + | + |
| $L^3$ | + | + | - | + |
| 1 | ++++ | ++ | +++ | +++ |
| 2 | +++ | ++ | ++ | ++ |
| 3 | +++ | +++ | +++ | +++ |
| 4 | +++ | +++ | +++ | +++ |
| 5 | +++ | ++++ | +++ | ++ |
| 6 | +++ | ++ | + | ++ |
| 7 | ++++ | +++ | +++ | +++ |
| 8 | +++ | +++ | +++ | ++ |
| 9 | ++++ | +++ | +++ | +++ |
| 10 | ++ | ++ | + | ++ |
| 11 | +++ | +++ | ++ | +++ |
| 12 | +++ | ++ | +++ | +++ |

a=*Escherichia coli*, b=*Pseudomonas aeruginosa*,
c=*Staphylococcus aureus* d=*Klebsiella pneumonae*
Inhibition zone diameter mm (% inhibition): +, 6-10 (27-45 %); ++, 10-14 (45-64 %); +++, 14-18 (64-82 %); ++++, 18-22 (82-100 %). Percent inhibition values are relative to inhibition zone (22 mm) of the most active compound with 100 % inhibition.

### Infrared Spectra

The bonding of the ligand to the metal ion was investigated by mainly comparing the infrared spectra of the free ligands with the spectra of their metal complexes (Table 3). The infrared spectra show:

a A positive shift of bands at 1515 and 613 $cm^{-1}$ due to skeletal modes of the pyrazine ring, indicating[19,20] co-ordination through pyrazine ring nitrogen.

b Insignificant shift of ν(C=O) at 1670 $cm^{-1}$ indicated that it is not co-ordinated to the metal atom.

c The spectra of all complexes indicated that a band at 1625 $cm^{-1}$ due to azomethine ν(C=N) linkage was shifted towards lower frequency by 5-10 $cm^{-1}$, respectively, indicating that the ligands are involved in co-ordination to the metal atom via the azomethine nitrogen.

d The new bands appearing in the spectra of metal complexes and not observed in the spectra of the ligands within 442-455 $cm^{-1}$, 375-386 $cm^{-1}$ and 510-525 $cm^{-1}$ assigned to M-O, M-S and M-N modes, respectively, indicated that the heteroatoms X are also involved in co-ordination.

These results however, indicated a structure of the type shown in Figure 2 but others could obviously be envisaged.

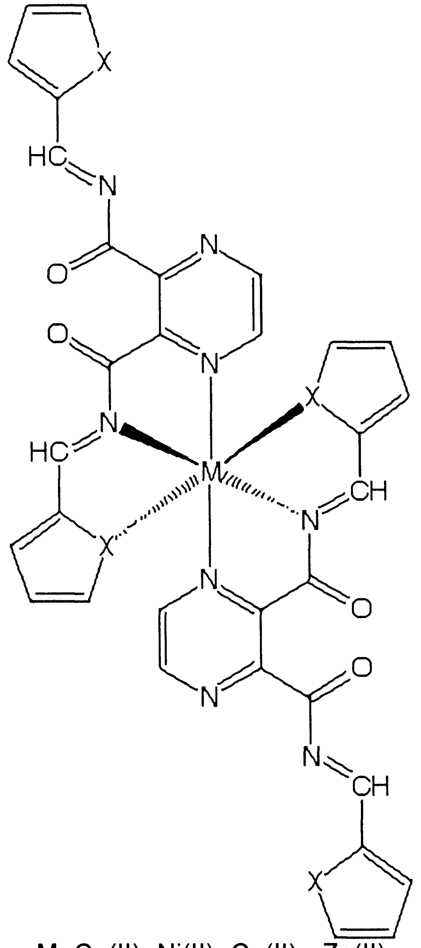

M=Co(II), Ni(II), Cu(II) , Zn(II)

**Figure 2.** Proposed structure for the metal(II) chelates

**Magnetic Moments**

The room temperature magnetic susceptibility measurements (Table 2) for the solid complexes lie within the range expected for octahedral geometry. Three unpaired electrons per Co(II) ion ($\mu_{eff}$ = 4.45-4.68 B.M), two unpaired electrons per Ni(II) ion ($\mu_{ef}$= 2.88-3.14 B.M) and one unpaired electron per Cu(II) ion ($\mu_{eff}$ = 1.55-1.82 B.M) suggesting[21-23] octahedral geometry for Co(II) and Ni(II) complexes and distorted octahedral geometry for for Cu(II) complexes. The observed magnetic susceptibilities have, in general, lower values than the expected moment which is probably due to antiferromagnetism arises via a superexchange mechanism.

### Electronic Spectra

Electronic spectra of the metal complexes are recorded in Table 2. The spectra of the cobalt chelates show three bands observed at 8715-9540 $cm^{-1}$, 17355-18125 $cm^{-1}$ and 29115-31235 $cm^{-1}$ which may be assigned to $^4T_1 \rightarrow {}^4T_2g$ (F), $^4T_1g \rightarrow {}^4A_2g$ (F) and $^4T_1g \rightarrow {}^4T_1g$ (P) transitions, respectively, and are suggestive[24,25] of octahedral geometry around the cobalt ion. Three bands observed at 9325-10132 $cm^{-1}$, 15820-17225 $cm^{-1}$ and 26375-27650 $cm^{-1}$ in the spectra of the nickel(II) chelates are due to spin-allowed transitions $^3A_{2g}(F) \rightarrow {}^3T_2g$ (F), $^3A_2g \rightarrow {}^3A_1g$ (F) and $^3A_2g \rightarrow {}^3T_1g$ (P), transitions, respectively, in an octahedral environment[26,27]. The copper(II) chelates show bands around the region 23155-23418 $cm^{-1}$ and 29450-30675 $cm^{-1}$.The lower energy band may be assigned to the transition $^2Eg \rightarrow {}^2T_2g$ as 10 Dq due to a distorted octahedral environment[27] and the band in the region 29450-30675 $cm^{-1}$ can be attributed to ligand metal charge transfer. The absorption spectra of Zn(II) complexes similarly, show a band at 13125-13514 $cm^{-1}$ due to d-d transitions in its distorted octahedral environment[28].

Based on the above evidences, it is proposed that cobalt(II) and nickel(II) complexes have an octahedral geometry (Figure 2) whereas Cu(II) and Zn(II) complexes have a distorted octahedral geometry in which the ligands behave as tridentate and accommodate themselves in such a way that a stable chelate ring is formed around the metal atom thus attains a stable configuration.

### Antibacterial Studies

The title ligands in comparison to their metal complexes were screened against bacterial species *Escherichia coli, Pseudomonas aeruginosa, Staphylococcus aureus and Klebsiella pneumonae* in order to determine their antibacterial properties. The antibacterial activity was tested at a concentration 30(g/0/01 mL in DMF using paper disc diffusion method.

The results of these studies reported in Table 5 showed that ligands and all their metal complexes are biologically active against one or more bacterial species and the metal complexes have been shown to be more antibacterial than the simple uncomplexed parent ligands.

### References

1 A.Masuko, I.Namura and Y.Saito, *Bull.Chem.Soc.Japan*,.**40**, 511 (1967).

2 Y.Nawata, H.Iwaski and Y.Saito, *Bull.Chem.Soc.Japan*, **40**, 515 (1967).

3 R.C.Paul, H.Arora and S. L. Chadha, *J.Inorg.Nucl.Chem.*,6, 469 (1970).

4 P.P.Singh, S.A.Khan and A.K.Sirivastava, *Ind.J.Chem.*, 12, 192 (1974).

5 A.D.Khamraev, *Zh.Strukt.Khim.*, **10**,1036 (1960).

6 J.R.Allan, H.J.Bowley, D.L.Gerrard, A.D.Patton and K.Turvey, *Inorg.Chim.Acta*, **132**, 41 (1987).

7 J.R.Allan, A.D.Patton, K.Turvey, H.J.Bowley and D.L.Gerrard, *Inorg.Chim.Acta*, **134**, 259 (1987).

8 S.Ali, M.Pervaz, A.Kalsoom and M.Mazhar, *Proc.Natl.Chem.Conf.*, **1**, 618 (1989).

9 C.L.Klein, C.J.O'Connor, R.J.Majeste and L.M.Trefonas, *J.Chem.Soc (Dalton Trans).*, 2419 (1982).

10 Z.H.Chohan and A.Rauf, *Synth.React.Inorg.Met-Org.Chem.*, **26**, 591 (1996).

11 Z.H.Chohan and A.Rauf, *Metal-Based Drugs*, **3**, 211 (1996).

12 Z.H.Chohan and H.Pervez, *Synth.React.Inorg.Met-Org.Chem.*, **23**, 1061 (1993).

13 Z.H.Chohan, S.K.A.Sherazi and M.Praveen, *Synth.React.Inorg.Met-Org.Chem.*, (in press).

14 Z.H.Chohan and S.Kausar, *Chem.Pharm.Bull.*, **40**, 2555 (1992).

15 Z.H.Chohan and A.Rauf, *J.Inorg.Biochem.*, **46**, 41 (1992).

16 D.H.Williams and I.Fleming,"Spectroscopic Methods in Organic Chemistry", 4th Ed, Mc Graw Hill, London (1989).

17 A.M.Shallary, M.M.Moustafa and M.M.Bekheit, *J.Inorg.Nucl.Chem.*, **41**, 267 (1979).

18 W.J.Geary, *Coord.Chem.Rev.*, **7**, 81 (1971).

19 C.Pelizzi and G.Pelizzi, *Inorg.Chem.Acta*, **18**, 39 (1976).

20 R.C.Aggarwal, N.K.Singh and R.P.Singh, *Inorg.Chem.*, **20**, 2794 (1981).
21 M.D.Glick and R.L.Lintvedt, *Prog.Inorg.Chem.*, **21**, 233 (1976).
22 A.B.P.Lever, *Inorg.Chem.*, **4**, 763 (1965).
23 E.K.Barefield, D.H.Busch and S.M.Nelson, *Quart.Rev.*, **22**, 457 (1968).
24 A.D.Liehr, *J.Phys.Chem.*, **67**, 1314 (1967).
25 R.L.Carlin,"Transition Metal Chemistry", Vol 1, Marcel Decker, New York, 1965.
26 A.B.P.Lever,"Inorganic Electronic Spectroscopy", 2nd Ed,Elsevier, New York, 1984.
27 D.W.Meek, R.S.Drago and T.S.Piper, *Inorg.Chem.*, **1**, 285 (1962).
28 C.J.Balhausen,"Introduction to Ligand Field",Mc Graw Hill, New York, 1962.

**Received: October 21, 1998 - Accepted: November 11, 1998 - Received in revised camera-ready format: November 26, 1998**

